# Functional repertoire of EV-associated miRNA profiles after lipoprotein depletion via ultracentrifugation and size exclusion chromatography from autologous blood products

**DOI:** 10.1038/s41598-021-84234-5

**Published:** 2021-03-12

**Authors:** Alexander Otahal, Olga Kuten-Pella, Karina Kramer, Markus Neubauer, Zsombor Lacza, Stefan Nehrer, Andrea De Luna

**Affiliations:** 1 Center for Regenerative Medicine, Department for Health Sciences, Medicine and Research, Danube University Krems, Krems an der Donau, Austria; 2 OrthoSera GmbH, Krems an der Donau, Austria; 3 Department of Sports Physiology, University of Physical Education, Budapest, Hungary

**Keywords:** Biological techniques, Molecular biology

## Abstract

Cartilage breakdown, inflammation and pain are hallmark symptoms of osteoarthritis, and autologous blood products such as citrate-anticoagulated platelet-rich plasma (CPRP) or hyperacute serum (hypACT) have been developed as a regenerative approach to rebuild cartilage, inhibit inflammation and reduce pain. However, mechanisms of action of these blood derivatives are still not fully understood, in part due to the large number of components present in these medical products. In addition, the discovery of extracellular vesicles (EVs) and their involvement in intercellular communication mediated by cargo molecules like microRNAs (miRNAs) opened up a whole new level of complexity in understanding blood products. In this study we focused on the development of an isolation protocol for EVs from CPRP and hypACT that can also deplete lipoproteins, which are often co-isolated in EV research due to shared physical properties. Several isolation methods were compared in terms of particle yield from CPRP and hypACT. To gain insights into the functional repertoire conveyed via EV-associated miRNAs, we performed functional enrichment analysis and identified NFκB signaling strongly targeted by CPRP EV miRNAs, whereas hypACT EV miRNAs affect IL6- and TGFβ/SMAD signaling.

## Introduction

Autologous blood derived products have already been used for more than a decade and increasingly gain popularity in regenerative medicine. A main application is the intra-articular injection into affected joints of osteoarthritis (OA) patients^1^, besides other treatments of muskuloskeletal conditions such as epicondylitis or rotator cuff injuries^2,3^. Platelet-rich plasma (PRP) is the most commonly used blood product for these conditions. The term refers to a fraction of whole blood, which is obtained after centrifuging a small amount of whole blood drawn from a peripheral vein in presence of an anti-coagulant such as citrate to obtain citrate-anticoagulated PRP (CPRP). A plethora of commercially devices are available to standardise PRP production^4^. Upon intra-articular injection, it is hypothesised that platelets are activated via contact with tissue factor and release a variety of growth factors and cytokines that contribute to tissue homeostasis and regeneration^5^. In contrast, serum-derived blood products have been developed including autologous conditioned serum (ACS) or hyperacute serum (hypACT). These fractions are obtained after extracorporeal coagulation of a small volume of fresh whole blood and squeezing out the soluble fraction of the fibrin clot. As for PRP, a variety of preparation devices exist that assist and standardise the production procedure. The rationale behind serum-derived blood products is to increase the concentration of natural anti-inflammatory molecules such as IL-1 receptor antagonist (IL1ra) released into whole blood after activation of blood coagulation^6–8^. Traditionally, research on the mechanisms of action of blood products has focused on growth factor and cytokine contents^9–11^.

Nevertheless, the discovery of extracellular vesicles (EVs) as novel components present in blood products and their recognition as relevant signaling players in tissue homeostasis^12^ have increased the level of complexity in understanding the effects of blood product therapies. EVs are a heterogeneous group of cell-derived membrane particles with a size of around 50 up to 2000 nm. They are classified by their biosynthetic origin whether they are intralumenal exosomes shed from multivesicular bodies, plasma membrane buds termed microvesicles released from the cell surface or apoptotic bodies derived from dying cells^12^. The approximate sizes of these particle types vary and size ranges overlap, thereby usually reducing the reasonableness of particle size as a specific analytic parameter. Once an EV has been generated it is almost impossible to determine its type as there are no molecular markers that uniquely identify a given EV subtype^13^. However, plasma membrane derived particles expose phosphatidylserine^14^ whereas higher amounts of tetraspanins such as Alix, CD9, or CD63 tend to be found in EVs originating from the cell interior^15^.

There is mounting evidence that EVs are the basis of regeneration in terms of cartilage recovery as shown for mesenchymal stem/stromal cell (MSC) based therapies of OA in vitro and in vivo^16–18^.  In these studies it was observed that beneficial effects were mediated by the secretome of MSCs, in particular EVs, rather than by cell-based mechanisms. EVs bring along a variety of signaling molecules such as miRNAs that might have a disease modifying effect in OA. However, as current research focuses mainly on MSC-derived EVs little is known about EV populations in blood products regarding chondroregenerative potentials. Although blood products pose a more direct approach of obtaining and applying autologous EVs while circumventing practical hurdles associated with cell-based therapeutics such as uncontrolled cell growth or graft rejection, besides regulatory hurdles^19^.

**Figure 1:**
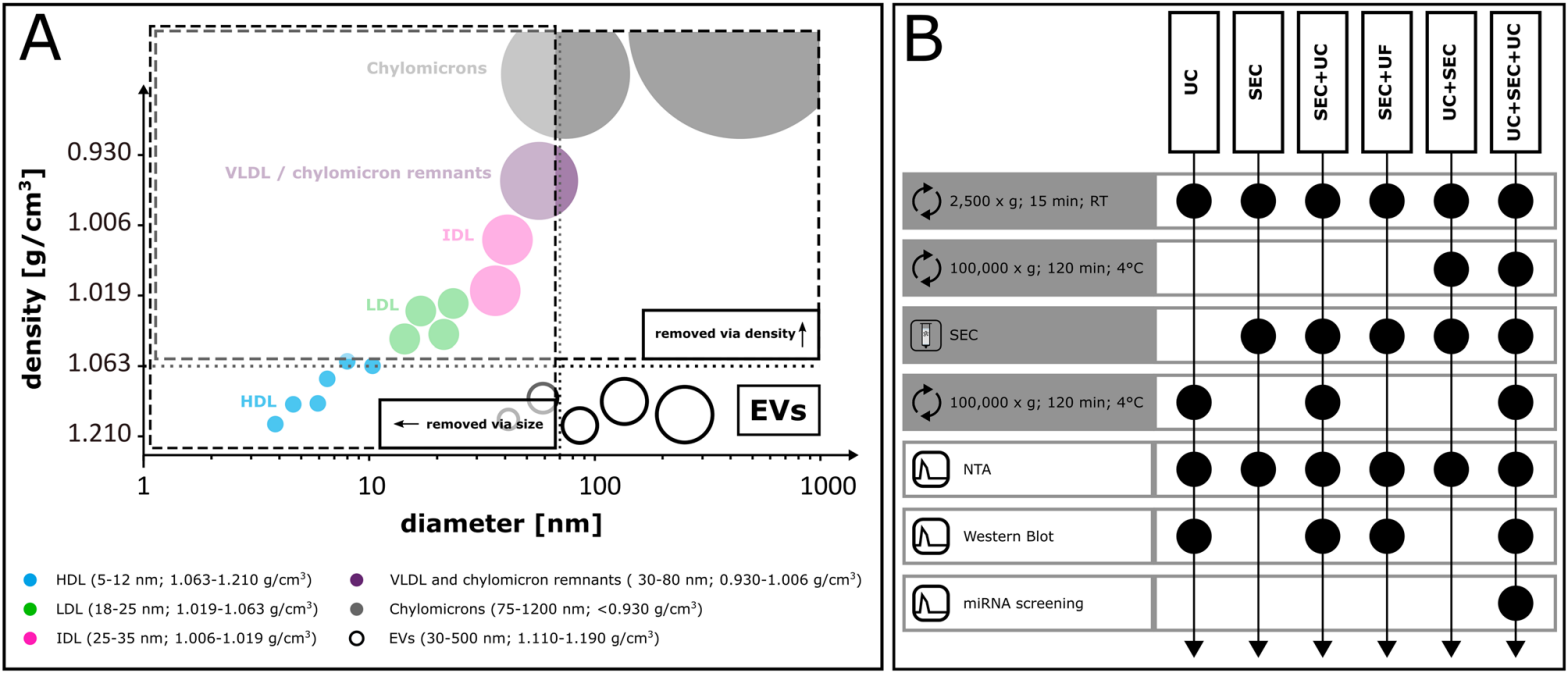
(**A**) Rationale behind using density- and size-based EV isolation techniques *in tandem* to deplete non-EV particles from plasma or serum (modified after^21^). (**B**) Overview of isolation procedures investigated in this study. Black dots indicate that the preparative step (grey row labels) or analytic step (white row labels) was a task in the procedure.

So far, a range of EV isolation techniques have been developed which employ either size- or density-based separation principles. Currently, the gold standard method is differential ultracentrifugation (UC)^20,21^, which enriches particles according to density. Limitations are the reported co-enrichment of lipoproteins and aggregates of soluble proteins that overlap in density with EVs^22,23^. To avoid aggregate formation and to enable gentle isolation of EVs, size exclusion chromatography (SEC) has been used to enrich EVs^24–28^, but it was observed that specific classes of lipoproteins with similar size as EVs were still present in the SEC fractions^29^. SEC dilutes particles, therefore concentrating steps have to be performed afterwards. This might employ UC or ultrafiltration (UF), a density- or again size-based method, respectively. However, performing two consecutive size-based methods *in tandem* like SEC for EV enrichment and UF for concentrating would prevent the depletion of lipoproteins co-isolated via SEC. UC depletes large lipoproteins with low buoyant density, but might co-pellet small dense lipoproteins. On the other hand, SEC could remove these small dense lipoproteins together with remaining soluble proteins, after large lipoproteins have been removed in a first step via UC (Fig. 1A). Therefore, we hypothesised that a combination of UC and SEC *in tandem* might provide a procedure to obtain highly pure EVs from plasma and serum without lipoprotein or soluble protein contamination. Furthermore, we aimed to outline the EV-associated miRNA species and their functional repertoire enriched in EV preparations from CPRP and hypACT to characterise expected functional associations, which might qualify isolated EVs suitable for cartilage regeneration and might explain disease modifying effects mediated by blood products in OA.

## Methods

### Preparation of blood products CPRP and hypACT

Blood products were prepared from human voluntary whole blood donations freshly drawn in house by approved personnel. We obtained informed consent from the donors and approval from the Ethics Committee of the Danube University Krems (ESC1020/2020). All experiments were performed in accordance with relevant guidelines and regulations. Eligibility criteria were age between 25 and 45 years, healthy at the time of donation according to a self-evaluation form asking for conditions such as diabetes, underweight or pregnancy as exclusion criteria. Donors were randomly selected from a pool of 4 female and 3 male donors. Blood was collected into citrate coated collection tubes (VACUETTE 9NC trisodiumcitrate 3.2%, Greiner BioOne, #455322) for preparing citrate-anticoagulated platelet-rich plasma (CPRP) or was drawn via the hypACT inject device developed by OrthoSera (Krems, Austria) to produce hyperacute serum (hypACT). Blood products were prepared as previously described^30^. In brief, CPRP was obtained from whole blood via a first round of centrifugation at 440 × *g* at room temperature (rt) for 10 min to pellet blood cells, transfer of the top fraction containing platelets and spinning this again at 1710 × *g* for 10 min at rt. Two-thirds of the supernatant were then discarded and the platelet pellet was resuspended in the remaining plasma. Preparation of hypACT involved a single round of centrifugation at 1710 × *g* for 10 min at rt. Afterwards, serum was squeezed from the fibrin clot that was formed in the device in the meantime. Blood products were processed immediately. CPRP and hypACT were prepared from the same donor in parallel for each isolation procedure. Due to limited sample volume per donation, blood products could not be used to perform different isolation procedures from the same blood product preparation.

### EV enrichment procedures

Freshly prepared blood products were pre-cleared from residual cells and cell debris via centrifugation at 2500 × *g* for 15 min at rt in 15 ml polypropylene tubes. The supernatant (S2 fraction) was used for subsequent enrichment procedures as outlined in Fig. 1B. Ultracentrifugation steps were conducted at 100,000 × *g* for 120 min at 4 °C in a MLA-80 fixed angle rotor (k-factor 29; 45,000 rpm). SEC-based separation was performed via pre-packed sepharose columns (iZON, qEVoriginal). PBS was sterile filtered prior to use (Sartorius, #16534) and was devoid of Ca^2+^ and Mg^2+^.

#### EV enrichment via ultracentrifugation (UC)

S2 fraction (2.5 ml) was transfered into polycarbonate ultracentrifugation tubes (Beckman-Coulter, #355647) and PBS was added to a final volume of 5 ml. Following balancing with PBS and ultracentrifugation, the EV pellet was resuspended in 200 µl PBS by gentle pipetting (P100 fraction). For western blotting, aliquots were taken directly after ultracentrifugation of the floating fat layer (fat fraction) and the cleared supernatant (S100 fraction). Samples were stored at − 80 °C until further analysis.

#### EV enrichment via size-exclusion chromatography and pooling via UC (SEC + UC)

After equilibrating the column with 10 ml PBS, aliquots of 500 µl of S2 fraction were loaded onto a SEC column. Columns were eluted with PBS. Collection of 1 ml fractions was started immediately after adding 14 ml elution buffer. After collecting 20 fractions, the column was washed with 1 bed volume (10 ml) PBS and stored at 4 °C. Fractions were stored at 4 °C for further analysis on the same day or frozen at − 80 °C and quickly thawed at 37 °C in a water bath. After performing nanoparticle tracking analysis (NTA) as described below to determine particle-rich fractions, corresponding fractions were pooled into polycarbonate ultracentrifugation tubes (Beckman-Coulter, #355647) and PBS was added to a final volume of 5 ml. After ultracentrifugation of pooled fractions 3–5, the EV pellet was resuspended in 200 µl PBS. Isolates were stored at − 80 °C until further analysis.

#### EV enrichment via UC and SEC *in tandem* and pooling via UC (UC + SEC + UC)

EVs were enriched from 2.5 ml S2 fraction as described above. The pellets were resuspended in 500 µl PBS before loading onto a SEC column and separating EVs from residual soluble proteins as outlined before. Afterwards, particle concentration was assessed in SEC fractions. Following pooling of fractions 3–5, particles were pelleted via ultracentrifugation and resuspended in 200 µl PBS by gentle pipetting.

#### EV enrichment via SEC and pooling via ultrafiltration (SEC + UF)

S2 fraction (500 µl) was separated via SEC as described above. The SEC fractions 3 to 5 (1 ml each) were pooled and ultrafiltrated at 10,000 × *g* and rt via a Vivaspin PES 100 kD cutoff filter (Sartorius, #VS0141). Particles were resuspended in 200 µl PBS by gentle pipetting and stored at − 80 °C.

### Nanoparticle tracking analysis (NTA)

Sizes and concentrations of particles in EV isolates or SEC fractions were determined via NTA using the particle analyzer ZetaView from Particlemetrix in scatter mode. The measurement procedure was done as previously described^31,32^. In brief, samples were diluted as required 1:20–1:1000 with PBS. Pre-acquisition camera settings were kept constant at sensitivity 80 and shutter 100. Videos were aquired at 11 positions each with 30 frames per second in one aquisition cycle. Video analysis was done with ZetaView 8.04.02 software using a minimum and maximum particle area of 5 and 1000, respectively. Mode size of particle size distributions was determined after smoothing of measured size profiles using a custom PHP-based implementation of the Savitzky-Golay algorithm as given in Supplementary Data 1^33^.

### Assessment of protein concentration

Total protein was determined via BioRad DC assay in a microplate format. 10 µl of undiluted or diluted sample as required were used. Samples were lysed with 10 µl RIPA buffer without protease inhibitors (Thermo Scientific, #89900) for 10 min at 4 °C to release EV protein content prior to performing the assay. A linear standard curve was generated from a serial dilution of bovine serum albumin (BSA) (Sigma-Aldrich, #A8022) ranging from 0 to 2 mg/ml.

### Western blot

EV suspensions (10 µg total protein) were mixed with 4 × LDS loading buffer (ThermoFisher, #NP0007) in presence (for Alix and ApoB100/48) or absence (for CD9, CD63 and ApoA1) of 100 mM dithiothreitol (DTT) and boiled at 95 °C for 10 min before loading onto pre-cast 10% or 4–12% gradient SDS-PAGE Tris-Bis gels (Invitrogen, # NP0301 and #NP0322). Proteins were blotted onto PVDF membranes (Invitrogen, #IB401002) under semi-dry conditions using an iBlot gel transfer device (Invitrogen, USA) for 26 min at 20 V. Successful transfer was checked by Ponceau S staining before destaining the membrane with deionised water and blocking in 5% non-fat dry milk in PBST (PBS + 0.1% Tween-20) for 1 h or overnight shaking at rt on an orbital shaker (15 rpm). After washing 3 times with PBST for 5 min at rt, membranes were incubated with primary antibodies diluted 1:1000 in 1% BSA/PBST against ApoA1 (Santa Cruz, #sc-376818), ApoB100/48 (Santa Cruz, #sc-393636), Alix (Cell Signaling, #2171), CD63 (BioLegend, #353005, clone H5C9) and CD9 (SystemBiosciences, #EXOAB-CD9A-1) for 1 h, followed by incubation with HRP-conjugated secondary antibodies (BioRad, #L9704446 and # L9704447) for 1 h. Proteins were detected via enhanced chemiluminscence (ECL) using WesternBright ECL substrate (Advansta, #K-12045-D20) and a ChemiDoc Reader (BioRad) equipped with PDQuest 7.4 software. Blot images were automatically uniformly white-corrected using the GIMP 2.8.22.

### RNA isolation and RT-qPCR

CPRP and hypACT were prepared as described above. Anonymised blood donor characteristics and isolated particle counts from 3 donors are given in Table 1. RT-qPCR based microRNA screening was performed by TAmiRNA GmbH (Vienna, Austria). Total RNA was extracted from blood products (CPRP or hypACT) as well as from EVs isolated via UC + SEC + UC, respectively, using the miRNeasy Mini Kit (Qiagen, #217004). Blood products and suspended EVs were transported frozen , thawed on ice and centrifuged at 12,000 × *g* for 5 min to remove cellular debris from blood products before RNA isolation. Each sample (200 µl) was mixed with 1000 µl Qiazol and 1 µl of a mix of 3 synthetic 22 nt long spike-in controls UniSp2, UniSp4 and UniSp5 (Qiagen, #339390) to monitor variability of RNA yield. After a 10 min incubation at rt, 200 µl chloroform were added to the lysates followed by centrifugation at 12,000 × *g* for 15 min at 4 °C. Precisely 650 µl of the aqueous phase were mixed with 7 µL glycogen (50 mg/ml) to enhance precipitation. Samples were transferred to a miRNeasy mini column, and RNA was precipitated with 750 µl ethanol followed by automated washing with RPE and RWT buffer in a QiaCube liquid handling robot. Finally, total RNA was eluted in 30 µL nuclease free water and stored at − 80 °C until further analysis. cDNA was synthesized using the miRCURY RT kit (Qiagen, #339340). Reaction conditions were set according to recommendations by the manufacturer suggesting 42 °C reverse transcription for 60 min, followed by 5 min at 95 °C for inactivation. In total, 18 µl of total RNA were used per 90 µl reverse transcription (RT) reaction. To monitor RT efficiency and presence of impurities with inhibitory activity, 4.5 µl of a synthetic 22 nt long RNA spike-in (cel-miR-39-3p, 0.002 fmol/µl) was included in the RT reaction. PCR amplification was performed in a 384-well plate format using a Roche LC480 II instrument and miRCURY SYBR Green mastermix (Qiagen, #339347). The Human miRNome Panel I was employed for screening miRNA expression (Qiagen, #339322). Each well contained a dried-down primer set specific for the individual reation. 4 spike-in control assays (UniSp2, UniSp4, UniSp5, cel-miR-39-3p), 3 interplate calibrators (IPC), 3 reference assays (U6snRNA, SNORD38B, SNORD49A) and blanks. In total, 12 plates were measured (isolated EVs and native blood product from CPRP and hypACT from 3 donors). cDNA was diluted 1:100 in 10 µl PCR reaction volume. Plates were incubated at 4 °C for 1 h prior to PCR amplification, using the following settings: 95 °C for 2 min, 45 cycles of 95 °C for 10 s and 60 °C for 60 s, followed by melting curve analysis. To calculate the cycle of quantification values (Cq-values), the second derivative method was used.

**Table 1:** Donor characteristics and sample details used for miRNA profile characterisation.

Donor	**D1**				**D2**				**D3**			
Gender	Female				Female				Male			
Age	29				42				28			
Fasting	No				No				No			
Blood product type	**CPRP**		**hypACT**		**CPRP**		**hypACT**		**CPRP**		**hypACT**	
Fraction type	**biofluid**	**EVs**	**biofluid**	**EVs**	**biofluid**	**EVs**	**biofluid**	**EVs**	**biofluid**	**EVs**	**biofluid**	**EVs**
Particles/ml blood product	n.d	1.03E + 09	n.d	5.46E + 08	n.d	7.86E + 08	n.d	8.20E + 08	n.d	9.26E + 08	n.d	2.98E + 08
Average mode particle size [nm] ± SD	n.d	179 ± 5	n.d	161 ± 26	n.d	155 ± 8	n.d	167 ± 5	n.d	199 ± 27	n.d	177 ± 10
Hemolysis indicator ΔCq(miR-23a—miR-451a) [Blondal et al. 2013]	0.82	1.60	10.23	2.02	0.13	0.60	10.23	0.88	-0.98	-1.14	9.93	3.50
Total miRNAs	332	275	332	215	327	267	324	200	336	285	310	184
Relevant miRNAs (Cq < 35)	275	149	254	49	282	150	251	64	273	185	227	39
Exluded miRNAs (Cq ≥ 35)	57	126	78	166	45	117	73	136	63	100	83	145
Undetected miRNAs	40	97	40	157	45	105	48	172	36	87	62	188
Estimated total miRNA copy number	4.37E + 10	6.27E + 07	1.02E + 11	3.64E + 06	4.46E + 10	9.58E + 07	9.05E + 10	5.00E + 06	2.21E + 10	2.26E + 08	1.85E + 10	1.68E + 06
Proportion of EVs carrying at least 1 miRNA	n.d	2.44%	n.d	0.27%	n.d	4.88%	n.d	0.24%	n.d	9.77%	n.d	0.23%
1 out of n EVs bears any miRNA	n.d	41	n.d	370	n.d	20	n.d	385	n.d	10	n.d	382
One single copy of a miRNA species per n particles, on average ± SD	n.d	2.59E + 04 ± 2.85E + 04	n.d	2.20E + 04 ± 1.64E + 04	n.d	1.75E + 04 ± 2.13E + 04	n.d	2.36E + 04 ± 2.03E + 04	n.d	1.30E + 04 ± 1.98E + 04	n.d	1.47E + 04 ± 8.62E + 03

Concentrations and mode sizes of EVs were determined in SEC fractions via NTA before final pooling via UC.

*n.d.* not determined.

### Profiling of miRNA expression

Analysis of PCR data was started by applying a Cq cutoff of 35 to select relevant hits. A miRNA was regarded as detected if it occurred in at least 2 out of 3 biological replicates per sample group. Next, the most stably expressed miRNA across sample groups was determined via NormFinder^34^ to use it as endogenous internal reference to normalise miRNA expression levels. The use of spike-in controls and/or the particle number for normalisation was dismissed as these parameters do not account for differences in RNA content between samples inherent to the biological materials. Cq values were then normalised to the Cq value of endogenous let-7a-5p in a given sample to obtain ΔCq values, which were ranked to perform rank analysis via Spearman rank correlation test. For quantitative assessment of miRNA enrichment or depletion in purified EVs, the fold change from ΔΔCq between EVs and blood product was calculated for each blood product. A ΔΔCq threshold of 1 or − 1 had to be exceeded to be considered a relevant change in expression. The significance of calculated fold changes was determined via multiple t-test while allowing a false discovery rate (FDR) < 0.05. Contamination due to hemolysis was assessed in all samples using the ratio of miR-23a-3p versus miR-451a^35^. Cq values of individual miRNAs were backcalculated to copy numbers relative to the average Cq (24,929) of cel-miR-39-3p of approximately 6.02 * 10^6^ spiked-in molecules corresponding to 0.001 amol/µl in 10 µl PCR reaction volume via the formula 6.02 * 10^6^ * 2^−(Cq-24.929)^ multiplied by a combined dilution factor of 166.6 (1.666-fold dilution of total RNA in RT mix and 100-fold dilution of cDNA in PCR reaction).

### Pathway analysis

Strong evidence gene targets for all miRNAs in EVs from CPRP or hypACT were obtained from mirTarbase 7.0 by selecting only targets verified by Western Blot, quantitative PCR or reporter assay from a database snapshot via the given SELECT statement: SELECT miRTarBase.[Target Gene] FROM miRTarBase WHERE (LOWER(miRTarBase.Experiments) LIKE “*reporter assay*”) Or LOWER(miRTarBase. Experiments) LIKE “*western Blot*” Or LOWER(miRTarBase.Experiments) LIKE “*qpcr*”) AND miRNA IN (<*miRNA list*>) GROUP BY miRTarBase.[Target Gene], where <*miRNA list*> is a placeholder for the respective miRNA query set. Targets were selected for all miRNAs identified in EVs from CPRP or hypACT, and enriched miRNAs in EVs, respectively. Next, STRING software v11 (https://string-db.org/) was used to identify enriched Gene Ontology (GO) Biological Processes terms and Reactome pathways among the proteins targeted by the miRNA sets^36–38^. Only GO and Reactome terms with highest confidence (≥ 0.9) were further investigated. To focus on highly relevant pathways, even more stringent selection criteria were applied by including only pathways having a confidence of at least 90% of the maximum confidence indicator found per group.

### Statistical analysis

Unless otherwise stated, data were tested with paired t-test using GraphPad version 8 to determine statistical significance which was accepted for *p* < 0.05. Two-way ANOVA and Sidak’s post hoc test were applied on NTA data, while RT-qPCR data were tested with multiple unpaired t-test accepting a false discovery rate (FDR) of 5% (q < 0.05). Discoveries were determined using the two-stage linear step-up procedure of Benjamini et al.^39^.

## Results

### Capacity of EV isolation methods for lipoprotein depletion

As EVs overlap in size and/or density with other particles such as lipoproteins present in blood and blood products, developing an isolation procedure that combines size- and density-based separation techniques could enable obtaining highly pure EV preparations (Fig. 1A). This is necessary to study effects mediated by EVs and mechanisms of action of EVs in blood products without confounding influences from other factors including lipoproteins and soluble proteins in plasma and serum. Several combinations of SEC and UC as given in Fig. 1B were evaluated in terms of particle yield and mode size via NTA.

**Figure 2:**
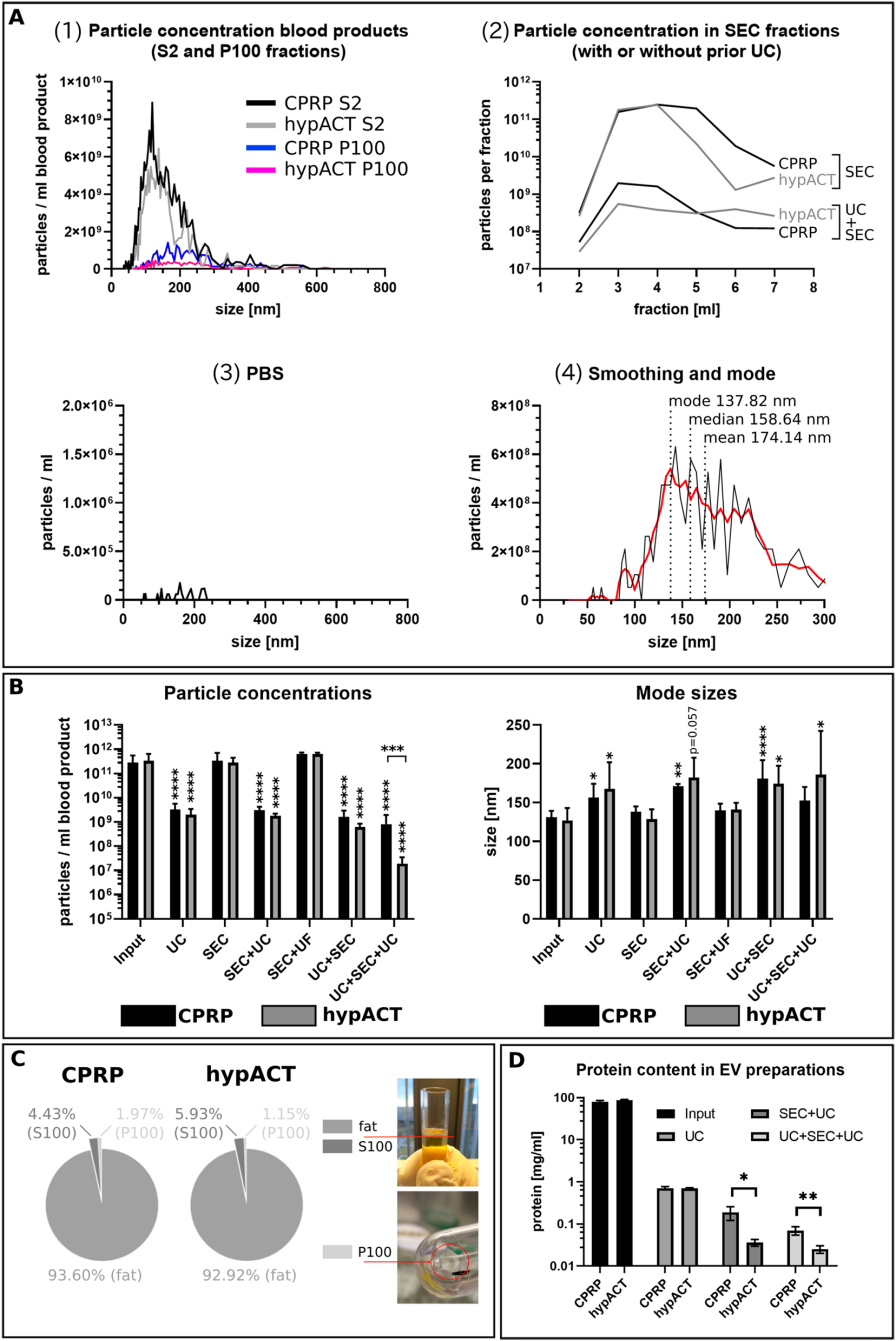
Particle concentration, mode sizes and protein concentrations of isolated EVs from CPRP and hypACT. (**A**) (1), representative size profiles of S2 and P100 fractions; (2), particle concentrations in SEC fractions with or without prior UC; (3), size profile of PBS control and (4), representative result of smoothing the raw size distribution via the Savitzky-Golay approach as described and comparison of mode, median and average particle size of a particle population as determined via NTA. (**B**) Particle concentrations and mode sizes in input and after indicated EV preparation procedures. Data from at least three biological replicates are given ± SD. (**C**) Evaluation of particle content after UC of S2 fraction in fat layer, S100 and P100 fractions, adapted from^21^. Data from three biological replicates are given as mean percentages of sum of particles in all fractions. (**D**) Protein concentration in input material and EV preparations obtained via the indicated procedures, normalised to input volume. Data from at least three biological replicates are given ± SD.*p < 0.05, **p < 0.01, ***p < 0.001, ****p < 0.0001.

Initial experiments comparing size profiles of CPRP or hypACT pre-cleared blood product (S2 fraction) revealed a dramatic difference in the total particle concentrations present in the input material before UC and the UC pellet (Fig. 2A). Next, particle concentrations of the same input material were determined after SEC and UC + SEC (Fig. 2A diagram 2). The huge particle concentrations in SEC fractions compared to UC + SEC fractions might indicate a tremendous amount of contaminating particles in SEC fractions, which could be lipoproteins based on the hypothesis that a prior UC step depletes these particles in the buoyant fat layer (Fig. 2C). The eluent (PBS) was essentially particle-free (Fig. 2A, diagram 3). The fourth diagram in Fig. 2A presents a representative result of data smoothing (red line) as described in the Methods section. The smoothing was used to denoise size distributions and to obtain a read out of mode size rather than average or median sizes, which tend to be higher than mode due to skewness of the particle size distribution. Figure 2B summarises particle concentrations found via different isolation methods, normalised to ml of input material. While input, SEC and SEC + UF (still) contained substantial amounts of lipoproteins, method combinations involving UC depleted more than 90% of particles each, compared to input. Determined particle concentrations were significantly different between isolation methods as assessed via one-way ANOVA and Dunett’s post-hoc test for CPRP (F(6, 29) = 36.29; *p* < 0.0001) and hypACT (F (6, 30) = 142.8; *p* < 0.0001). However, concentration differences between blood products for a given method were not significant, except for UC + SEC + UC (*p* < 0.001). Interestingly, mode sizes of smoothed particle size distributions revealed significant increases in mode size of particles obtained after methods involving UC compared to input as determined via one-way ANOVA and Dunnett’s post hoc test for CPRP (F (6, 29) = 6.725; *p* = 0.0002) and hypACT (F (6, 30) = 3.345; *p* = 0.0121) (Fig. 2A). In CPRP, UC (*p* = 0.019), SEC + UC (*p* = 0.007) and UC + SEC (*p* < 0.0001) were significantly different, respectively. This pattern was essentially recapitulated in hypACT for UC (*p* = 0.048), SEC + UC (*p* = 0.057), UC + SEC (*p* = 0.031) and UC + SEC + UC (*p* = 0.019), although with lower statistical significances. A buoyant layer, termed fat layer, contained the majority of particles after UC (Fig. 2C). Only 1–2% of all particles in blood products were found in UC pellets (P100). As UC was considered to deplete lipoproteins from input, which can be smaller than EVs (Fig. 1A), and a UC pellet contains enriched EVs, the mode size increased in EV preparations when the majority of non-EV particles was depleted which would cover up the true mode size of an EV size distribution as in input, SEC and SEC + UF. For any isolation method, mode sizes of particles were not significantly different between blood products (Fig. 2B). The protein concentration of EV preparation decreased with increasing number of isolation methods performed *in tandem*. The UC pellets contained 0.71 ± 0.06(SD) mg/ml or 0.69 ± 0.03(SD) mg/ml protein normalised to the respective input materials CPRP S2 (79.72 ± 5.88(SD) mg/ml) and hypACT S2 (86.56 ± 3.46(SD) mg/ml), respectively. After pooling SEC fractions via UC (SEC + UC), pellets contained 0.19 ± 0.07 (SD) mg/ml or 0.04 ± 0.01(SD) mg/ml protein, while UC + SEC + UC pellets yielded 0.07 ± 0.02(SD) mg/ml or 0.03 ± 0.01(SD) mg/ml protein for CPRP or hypACT, respectively. Each protein concentration represents the amount of protein that was isolated via the respective method per ml of input material (Fig. 2D).

### Protein marker profiling

**Figure 3:**
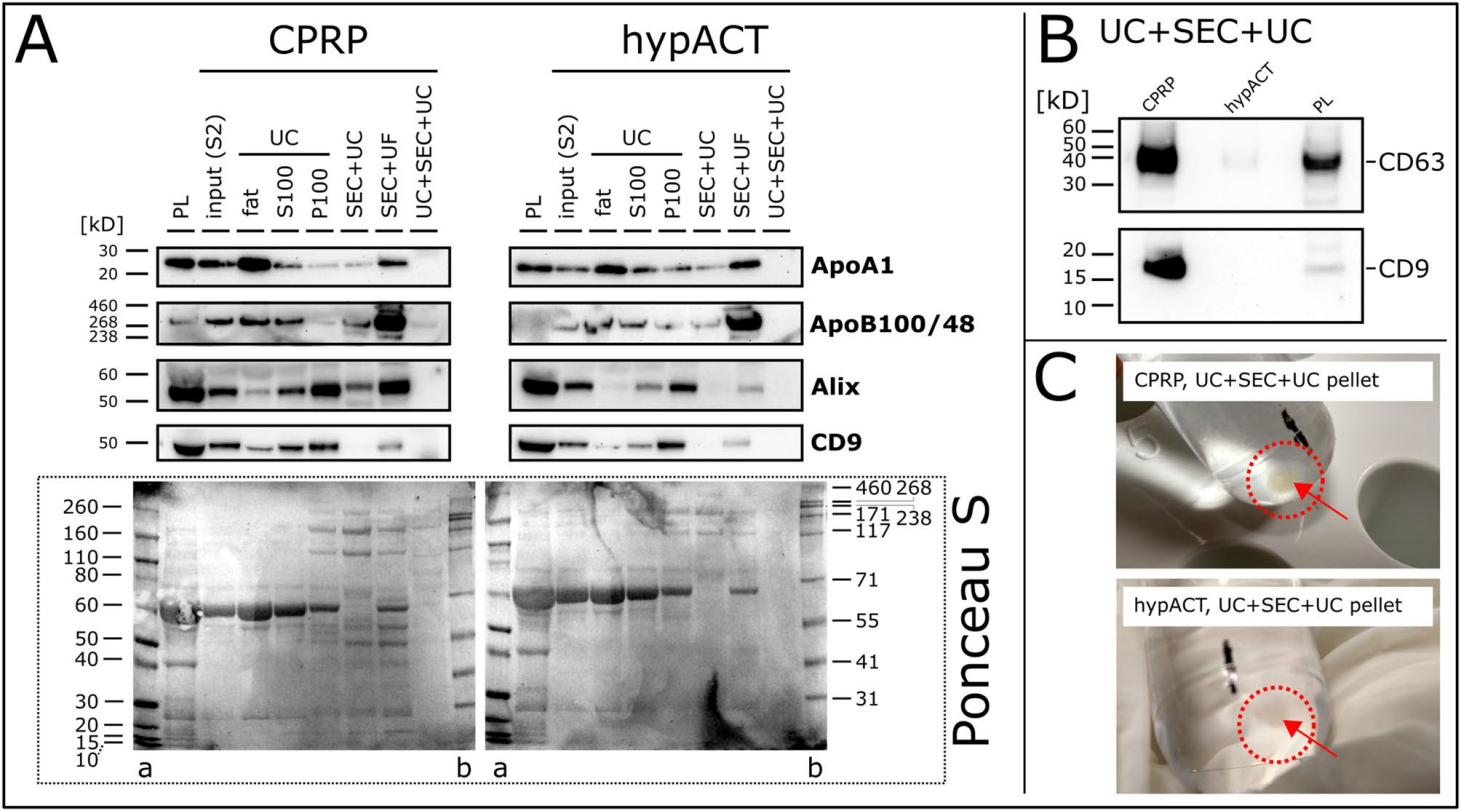
Western Blot of lipoprotein (ApoA1, ApoB100/48) and EV markers (Alix, CD9, CD63). (**A**) 10 µg protein was loaded per lane (except for PL, 20 µg) and membrane Ponceau S stained after transfer is shown. (**B**) Detection of CD63 and CD9 (80 µg per lane, except for 20 µg for PL) in UC + SEC + UC pellets. (**C**) UC + SEC + UC pellets obtained after removing supernatant. PL: platelet lysate, S2: plasma or serum supernatant after 2500 × *g* centrifugation, S100: supernatant after 100,000 × *g* centrifugation, P100:.pellet after 100,000 × *g* centrifugation, fat: buoyant layer after ultracentrifugation, a: marker (Invitrogen, #LC5800), b: marker (Invitrogen, #LC5699). Full-length blots/gels are presented in Supplementary Data 2.

As NTA in scatter mode is limited to detect particles without further characterisation, Western Blot analysis of EV preparations via different isolation methods was performed to monitor the recovery of EV markers Alix, CD9 and CD63, as well as the depletion of lipoproteins Apolipoprotein A1 (ApoA1) and Apolipoprotein B100 and its truncated variant B48 (ApoB100/48) found in high density lipoproteins (HDL) and (very) low density lipoproteins ((V)LDL) particles, respectively. As shown in Fig. 3A, ApoA1 was depleted below detection limit from CPRP and hypACT EVs via UC + SEC + UC, while the first UC pellet (P100) as well as SEC + UC co-pelleted ApoA1. Similarly, ApoB100/48 was depleted via combined density- and size-based isolation. As expected, SEC + UF yielded preparations with concentrated ApoB100/48, which is co-isolated via SEC. Nevertheless, also ApoA1 was enriched via SEC + UF. Unexpectedly, while UC pellets (P100) contained substantial amounts of Alix and dimeric CD9 (around 50 kDa), SEC + UC and UC + SEC + UC caused a dramatic or complete loss of signal, although same protein concentrations were loaded into each lane. When loading eightfold protein amount, monomeric CD9 (predicted molecular weight 25.42 kDa, Uniprot-ID P21926) was detected in UC + SEC + UC EVs for CPRP, but still absent for hypACT EVs. In addition, the membrane was probed for another EV marker, CD63, and a weak signal was detectable for hypACT EVs (Fig. 3B). Reduced samples were probed for Alix as well, however, Alix was neither detected in CPRP or hypACT EVs (data not shown). Nevertheless, this shows that there is no complete loss of EV marker proteins via UC + SEC + UC. Ponceau S stained membranes showed very faint, but visible, protein bands for UC + SEC + UC EVs, however, the majority of bands might be below the detection limit of Ponceau S stain, especially for hypACT (Fig. 3A).

### Blood product-derived purified EVs have disctinct miRNA profiles

**Figure 4:**
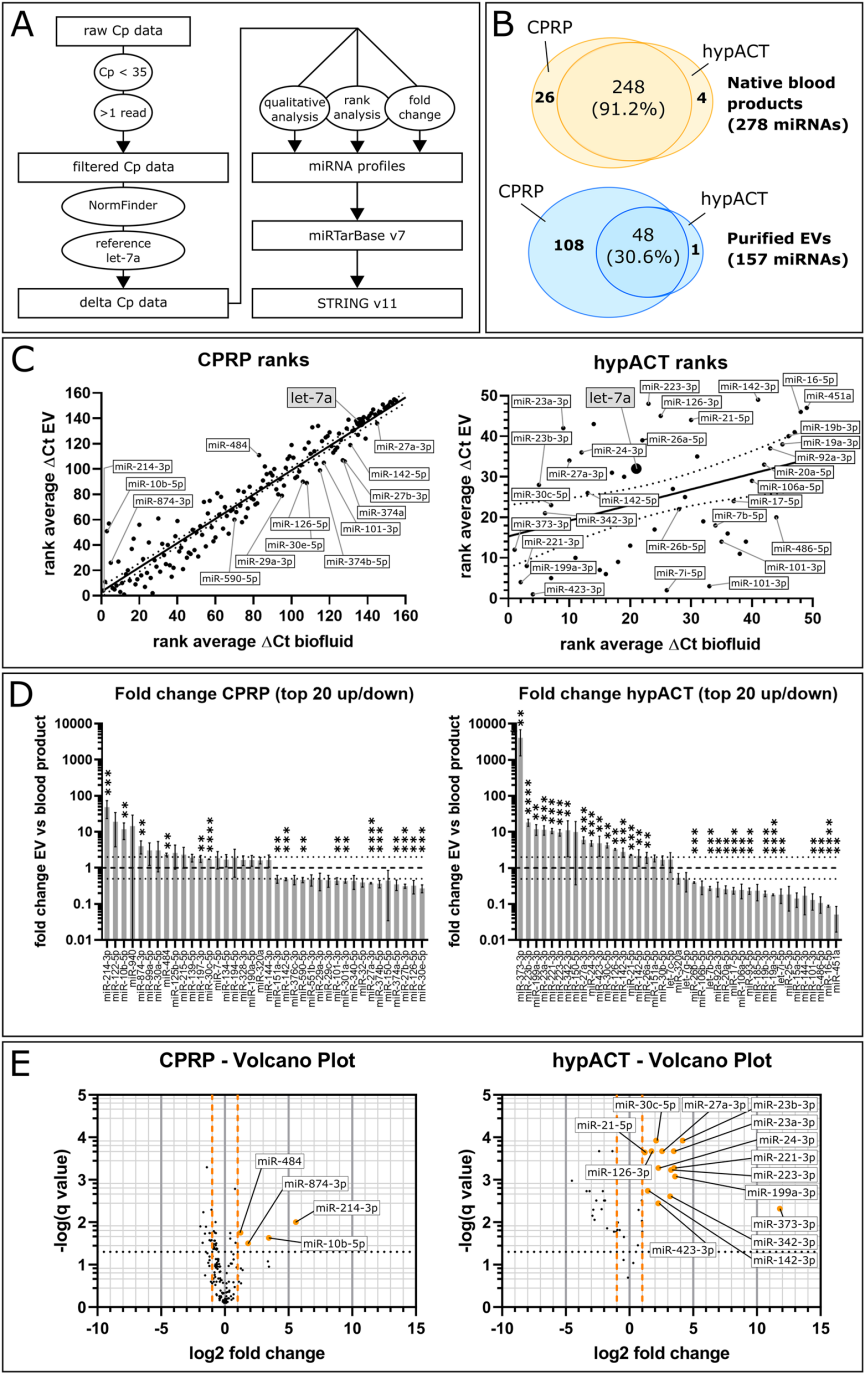
miRNA profiling. (**A**) RT-qPCR data analysis workflow. (**B**) Qualitative assessment of number of miRNA species identified in blood products and purified EVs. (**C**) Rank analysis of relative abundance of identified miRNAs in blood products and EVs for CPRP and hypACT, 156 and 49 miRNA species, respectively. Higher rank represents higher relative abundance. Spearman rank correlation resulted in r = 0.9592 (0.9439–0.9704 95% CI) for CPRP and r = 0.3879 (0.1113–0.6087 95% CI) for hypACT with *p* < 0.001 and *p* = 0.006, respectively. (**D**) Quantitative assessment of miRNA fold changes normalised to let-7a. Dotted black lines indicate relevance thresholds (twofold enrichment or 0.5-fold depletion). (**E**) Volcano plot of significantly enriched miRNAs. Black dotted line indicates significance threshold, orange dashed lines are relevance thresholds. Statistical significance (**D**,**E**) was assessed via multiple t-test accepting a false discovery rate of 5% (q < 0.05). Black dotted line indicates significance threshold, orange dashed lines are relevance thresholds. **p < 0.01, ***p < 0.001, ****p < 0.00001.

To determine EV miRNA cargo profiles in CPRP and hypACT, full blood product and purified EV preparations from 3 donors each were screened for a panel of 372 miRNAs via a reverse transcriptase quantitative PCR (RT-qPCR)-based assay in 384-well microplate format. Sample details are given in Table 1, the full miRNA panel is given in Table S1. The data analysis flow chart is shown in Fig. 4A. The efficiency of RNA isolation, reverse transcription and PCR showed low variability across samples (Fig. S1). Relevant data on EV isolation for miRNA assessment was deposited in the EVTRACK database, accession number EV190086. In total, 274 or 252 distinct miRNAs were detected in CPRP or hypACT blood products, respectively, whereas 156 or 49 miRNAs were found in purified EVs from CPRP or hypACT. The Venn diagrams (Fig. 4B) show an overview of the numbers of miRNAs found in blood products and EVs from CPRP and hypACT, while outlining the number of shared miRNAs between sample groups. The qualitative assessment revealed 26 miRNAs specific to CPRP and 4 miRNAs specific to hypACT when comparing blood products. The 26 CPRP blood product miRNAs were not found in CPRP EVs, neither 3 out of 4 miRNAs from hypACT blood product in EVs, except for miR-373-3p (Fig. 4B). CPRP and hypACT blood products shared 248 out of 272 miRNAs (91.2%), whereas the EV preparations had 48 out of 157 miRNAs in common (30.6%). The detection rates of miRNAs per sample were determined as the number of detected miRNAs (Cp < 35) in proportion to the whole 372 miRNA test panel (Fig. S1C). According to one-way ANOVA and Tukey’s post hoc test (F(3,8) = 148.7; *p* < 0.0001) they were significantly lower in EVs purified from hypACT or CPRP compared to the respective native blood product (q = 23.39 or 13.95, respectively). In addition, there were significantly more miRNAs detected in CPRP than in hypACT EVs (q = 13.39). Interestingly, similar amounts of miRNA species were found in both blood products (q = 3.952). Screening the raw Cq values for miRNA expression levels via NormFinder^34^ identified miRNA let-7a-5p as most stably expressed miRNA with stability value of 0.194. In the following, the Cq values were normalised to let-7a-5p in each sample to obtain ΔCq values. Average ΔCq values were assigned ranks to perform rank analysis of relative abundance changes between blood product and purified EVs in CPRP or hypACT (Fig. 4C). A higher rank indicates a higher relative abundance. Generally, the rank correlation observed for relative miRNA abundances in CPRP and hypACT resulted in Spearman correlation coefficients of 0.9589 (0.9435–0.9702 95% CI) and 0.3879 (0.1113–0.6087 95% CI), respectively (Fig. 4C). Linear regression analysis quantified the goodness of fit with R^2^ = 0.9201 in contrast to R^2^ = 0.1495 for CPRP and hypACT, respectively (Fig. 4C).

Quantitative enrichment analysis via calculating the fold change of miRNA abundances in purified EVs compared to blood products identified miR-214-3p as most enriched in CPRP EVs followed by miR-10b-5p, miR-874-3p and miR-484, whereas the amount of miR-373-3p increased strongest in hypACT EVs followed by miR-23b-3p, miR-199a-3p, miR-23a-3p, miR-221-3p, miR-223-3p, miR-342-3p, miR-27a-3p, miR-24-3p, miR-423-3p, miR-30c-5p, miR-126-3p, miR-142-3p, miR-21-5p and miR-142-5p (Fig. 4D). The significances of enriched miRNAs are highlighted in a volcano plot (Fig. 4E).

To visualise clusters of enriched or depleted miRNAs in all samples of all 3 donors, a heat map of miRNAs present in EVs from CPRP or hypACT or blood product was constructed (Fig S2). Clusters of miRNAs appear which were overrepresented in EVs from CPRP (cluster 1 and 2, miR-122-5p, miR-125b-5p, miR-214-3p, miR-10b-5p, miR-652-3p and miR-874, miR-840, miR-99a) and hypACT (cluster 3 and 4, miR-30b-5p, miR-150-5p, miR-342-3p, miR-30c-5p, miR-373-3p and miR-126-3p, miR-27a-3p, miR-24-3p, miR-223-3p, miR-23a-3p, miR23-b-3p). Interestingly, a large cluster (cluster 5) was evident in hypACT blood product including miRNAs miR-451a, miR-16 and miR-92 indicating contamination with erythrocytes^40^. Therefore, all samples were tested for hemolysis by calculating a ΔCp of miR-23a-3p and miR-451a as hemolysis indicator as reported elsewhere^35^. Values are given in Table 1. A ΔCp of 5 or more is indicative of erythrocyte miRNA contamination. An average ΔCp of 10.13 ± 0.17 (SD) was determined in hypACT blood product indicating strong hemolysis in these samples. In contrast, the hemolysis indicator stayed below 5 and took values of 2.13 ± 1.31 (SD), -0.01 ± 0.91 (SD) and 0.35 ± 1.37 (SD) for hypACT EVs, CPRP blood product and CPRP EVs, respectively. This underlines the absence of erythrocyte miRNAs in EV samples, which is an indicator of the absence of miRNAs potentially released from the cytoplasm of residual blood cells present in blood products upon freezing samples for transport. This is also presented on the heat map (Fig S2, cluster 5).

To determine how many miRNA sequences were found per particle, the copy numbers of individual miRNAs were estimated relative to spiked-in cel-miR-39-3p as described above. Then, copy numbers of all identified miRNAs in a given EV preparation were summed up and divided by the number of particles detected in the sample. Data are given in Table 1. For CPRP EVs, 5.69% ± 3.74% (SD) of all particles contained on average 1 miRNA molecule. This result implicates that probably a part of this proportion might contain zero miRNAs while others carry 2 or more miRNA molecules of any identified miRNA species or combination of miRNA species. On the other hand, the miRNA-bearing EV proportion in hypACT EVs amounted to 0.26% ± 0.01% (SD). That means that 1 out of 24 CPRP EVs ± 16 (SD) carried any one miRNA, while any one miRNA was present in 1 out of 379 hypACT EVs ± 8 (SD).

### Functional repertoire of miRNA populations in UC + SEC + UC purified EVs

Querying miRTarBase v7 with all miRNAs identified in EVs from CPRP or hypACT for experimentally validated target genes resulted in 2033 or 1042 verified targets, respectively. The 4 and 14 miRNAs significantly enriched in CPRP or hypACT EVs (Fig. 4C,D) were found to target 88 and 548 verified targets.

Pathway analysis via STRING found 640 reactome pathways and 3731 GO biological processes with FDR < 0.05 targeted by all CPRP EV miRNAs. Among those, 13 GO biological processes and 10 Reactome pathways were of highest confidence (> 0.9) and with an FDR < 0.001. Pathway analysis for all hypACT EV miRNAs resulted in 597 reactome pathways and 3722 GO biological processes with FDR < 0.05. Interestingly, 262 GO biological processes and 107 Reactome pathways remained after applying highest confidence and FDR < 0.001 filter constraints on the data set. Although hypACT EVs contained only around 30% of miRNA species compared to CPRP EVs, more than tenfold as much significant pathways and processes are targeted by hypACT EV miRNAs. Full list of highest confidence GO terms and Reactome pathways are given in Table S2. Targets returned by miRTarBase, but not considered for STRING analysis, are PDK1, PDK2, CACNA1C, ZNF763, PLAC8, SLC11A2, TAC1, XIST, WDR4, TFAM, UCP2, TSPYL2, TTN, TBR1, ARTN, BASP1. These were manually excluded from the query list, based on their associated functions not directly related with or not being specific to cartilage, to meet the input limit for STRING of 2000 protein identifiers.

**Figure 5:**
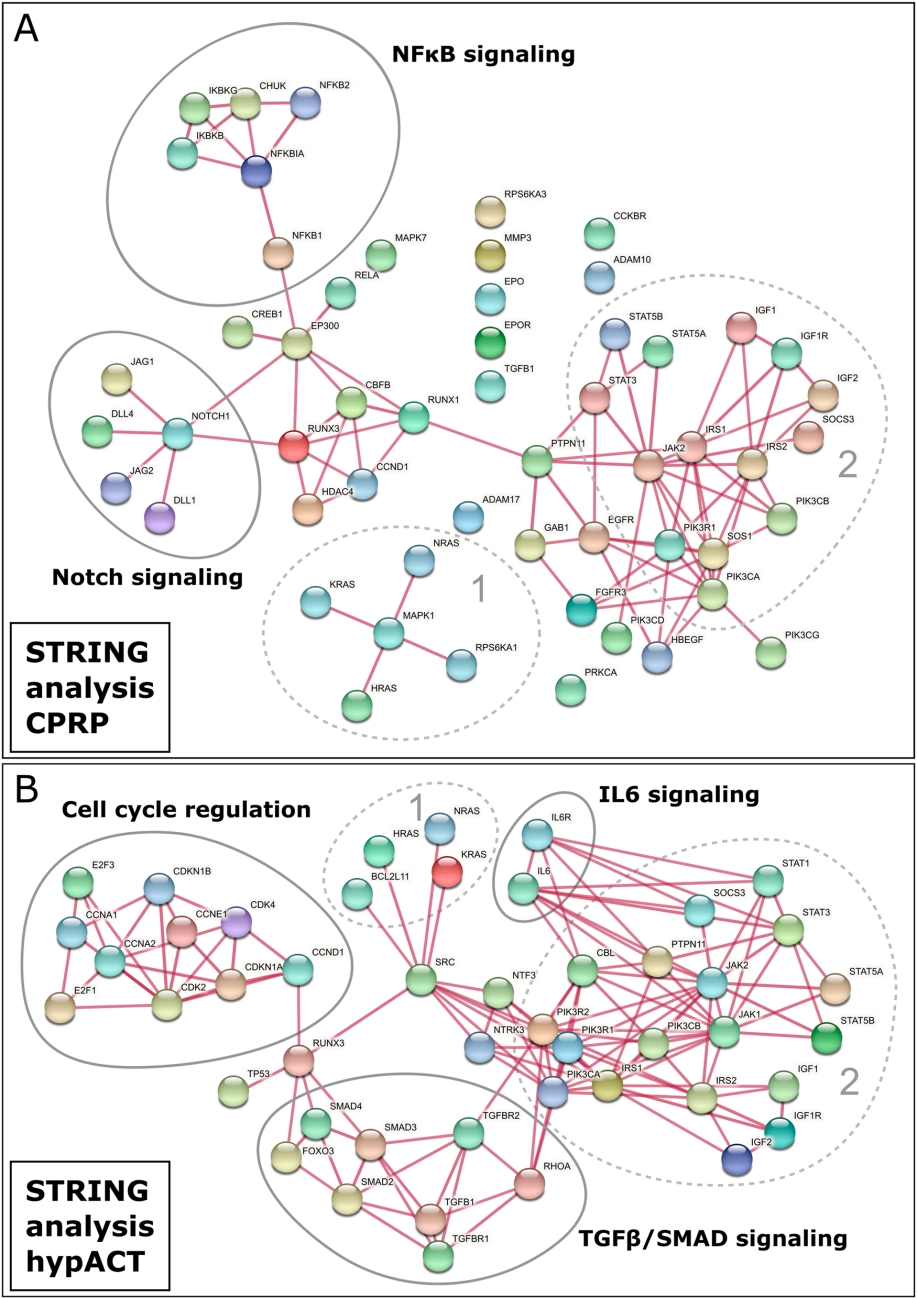
Physical STRING networks of miRNA targets in high confidence Reactome pathways for CPRP and hypACT EVs. Clusters 1 and 2 are found among miRNA targets for EVs from either blood product. (**A**) NFκB and Notch signaling are specifically targeted by CPRP EVs, while (**B**) TGFβ/SMAD signaling, IL6 signaling and cell cycle regulation are high confidence targets of hypACT EVs. Line thickness represents > 0.9 confidence of interaction.

Visualising 60 and 336 unique targets from the identified significant pathways in CPRP and hypACT EV miRNAs in gene clusters via STRING to outline shared nodes was hampered by their large number for hypACT in particular. Therefore, more stringent selection criteria were applied. A Reactome term had to reach a confidence of at least 90% of the maximum strength indicator of all terms with FDR < 0.001, resulting in a minimum strength of 0.891 or 1.161 for CPRP and hypACT, respectively. This yielded 9 Reactome terms and 13 GO biological processes for CPRP, while 14 Reactome terms and 11 GO biological processes were obtained for hypACT as given in Table S3. The GO enrichment resulted in biological processes related with regulation of apoptosis for CPRP EVs, while mainly cell-fate and differentiation related processes were found among targets of hypACT EV miRNAs. For the genes involved in Reactome pathways, STRING networks were generated from 55 and 47 Reactome gene targets for CPRP and hypACT EV miRNAs, respectively (Fig. 5A,B). Clusters involving HRAS/NRAS/KRAS were common to EV miRNAs from CPRP and hypACT as well as a cluster including insulin- and JAK/STAT-driven signaling (Fig. 5A,B, clusters 1 and 2). Clusters specific to CPRP EV miRNAs regulate NFκB and Notch/Delta signaling, while hypACT EV miRNAs target TGFβ/SMAD and IL6 signaling as well as cell cycle regulation.

**Figure 6:**
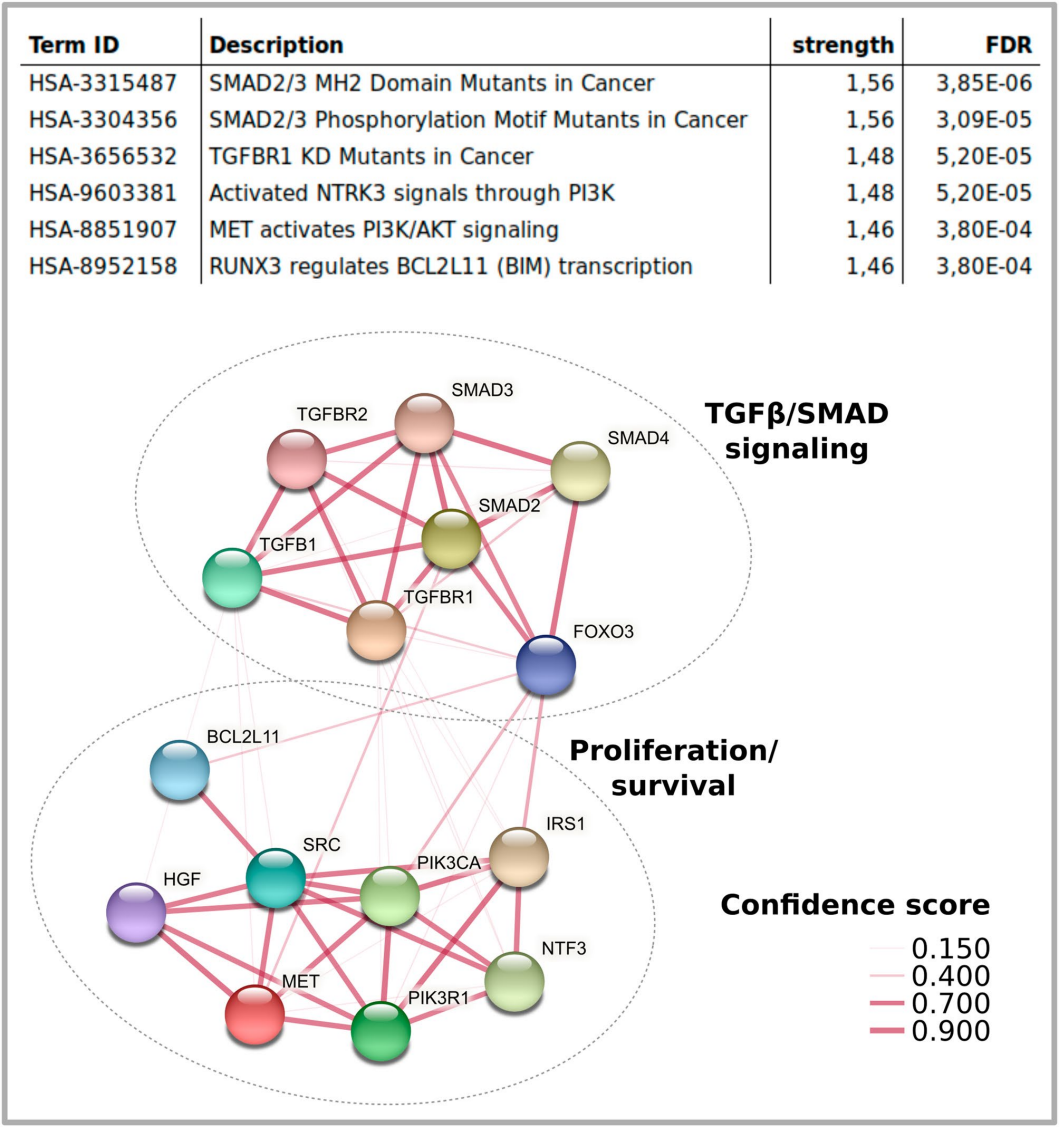
Maximum confidence gene targets of enriched miRNA in hypACT EVs. Reactome pathways presented as physical STRING clusters, line thickness indicates confidence of interaction.

To determine which paythways are increasingly targeted by the identified enriched miRNAs in CPRP and hypACT EVs (Fig. 4E), strong evidence miRNA targets were selected from mirTarBase and subjected to Reactome pathway enrichment analysis as before. While no pathways met the significance criteria of FDR < 0.001 and 90% maximum confidence for CPRP EV miRNAs, 6 Reactome terms were identified for enriched miRNAs in hypACT EVs totaling up to 15 gene targets as shown in Fig. 6. These targets formed two distinct clusters affecting proliferation and TGFβ/SMAD signaling.

## Discussion

Biofluids like plasma or serum are very complex mixtures, therefore studying mechanisms of action of single or a group of components like EVs and their cargo imposes challenges in terms of isolation and purification. It is known that CPRP and hypACT also contain pro-inflammatory cytokines like IL1β^8^ that would counterbalance regenerative effects mediated by EV-associated miRNAs. Since each EV isolation method has limitations and co-purifies particles and molecules that contaminate EV isolates such as lipoproteins, which overlap in size or density with EVs (Fig. 1), new protocols need to be developed in order to obtain pure EVs from blood products to study their immediate therapeutic effects.

For this reason, this work investigated combined density- and size-based isolation (UC and SEC) *in tandem* to obtain highly pure EV preparations devoid of soluble proteins and non-EV particles including lipoproteins. Earlier reports worked on the feasibility of this EV enrichment combination^41^, but did not investigate residual lipoprotein contaminations. Oxidised low density lipoprotein (ox-LDL) is found in blood and therefore in blood derived products^42^. This lipoprotein derivative can act pro-inflammatory^43,44^ via induction of IL1β release^45^ and could interfere with diseased cartilage upon blood product therapy. Chondrocytes express Toll-like receptors (TLRs)2 and TLR4 especially in OA which was associated with increased cartilage degradation and elevated inflammation^46–48^. TLR4, which is a receptor for ox-LDL, promotes pro-inflammatory macrophage differentiation and is involved in the onset of inflammatory conditions^49–51^. Similarly, TLR4 mediates IL1β expression in chondrocytes in presence of ox-LDL^52^. In addition, OA chondrocytes express lectin-like oxidised low-density lipoprotein receptor (LOX-1), another receptor for ox-LDL, and loss of LOX-1 protects against age-related knee OA via preventing VEGF-mediated metalloproteinase expression^53,54^. These findings highlight the relevance of ox-LDL in OA disease progression and the potential for improvement during production of autologous blood products and derivatives such as EV preparations. Beside adverse effects of LDL, HDL can bind pro-inflammatory miRNAs^55^, that might be taken up by OA chondrocytes and aggravate disease. There has also been a report on synovial inflammation driven by elevated levels of the HDL receptor scavenger receptor B1 (SR-BI or CD36), which was also found to be stronger expressed in OA cartilage driving chondrocyte hypertrophy, which might promote OA progression when taking up elevated amounts of HDL^56,57^. Therefore, also removal of HDL from EV preparations might be of interest especially when tracking down the regenerative potential of EVs.

A striking observation was that only 1–2% of all detected particles in plasma or serum can be found in a UC pellet (Fig. 2A,C) and that non-pelletable particles floating in a fat layer by far outnumbered EVs. In addition, the proportion of lipoproteins co-isolated via SEC was tremendous. Similar to an earlier report^58^, we observed an increase in particle size pre- and post-UC samples (Fig. 2B). Depletion of lipoproteins might provide an explanation for this shift in mode size of the whole particle populations, because lipoprotein-containg samples showed a size distribution with lower mode size that covered up size distributions of lipoprotein-depleted EV preparations (Fig. 2A).

Although, UC was reported to promote aggregate formation and to impose damage to EVs^59^, it is a gold standard method for EV enrichment^20^. However, to decrease viscosity of plasma and serum which is inversely proportional to sedimentation efficiency^58^, S2 fractions were diluted 1:1 with PBS in this study to reduce internal friction and to lower potential EV damage when EVs travel along the sedimentation path during centrifugation.

While we were able to show substantial depletion of lipoprotein content with UC + SEC + UC, this combination method resulted in considerable losses of EV marker signals on Western Blot (Fig. 3). This effect was obviously caused by concentrating SEC fractions via UC, because concentrating via UF recovered clearly detectable amounts of Alix and CD9. This might indicate that the concentration step was too short to pellet all the material from the pooled SEC fractions, although the final UC run lasted for 2 h. On the other hand, UC protocols often include washing steps for plasma and serum EVs without reporting similar effects in terms of particle damage or loss^20,60,61^, therefore EV damage due to UC is also less likely an explanation. More preparative steps might come at a cost in terms of EV yield, so the concentration of Alix or CD9 might have been simply too low for detection via Western Blot. To overcome this, we loaded eight-fold protein amount and probed for CD9 and CD63 and showed that these two EV markers are strongly present in CPRP EVs, in contrast to hypACT EVs. This might be explained by CD63^+^ and/or CD9^+^ platelet derived EVs contributing to and being partially consumed in blood coagulation in the hypACT device. As no protease inhibitors were used during the procedure with respect to a future clinical application of the EVs, residual protease activity can not be ruled out in hypACT^62^ that might have decreased epitopes below detection limit. Nevertheless, these samples were used for miRNA analysis.

When determining EV-associated miRNA cargo profiles, a potential contribution of other sources for extracellular RNA has to be considered^63^. By using SEC, it was expected that free-floating or Ago2-associated miRNAs in plasma or serum were separated from EVs based on the small size of Ago2^64^. The depletion of miR-16-5p and enrichment of miR-21 in hypACT EVs which were reported to be preferentially associated with Ago2 or EVs^65,66^, respectively, confirms our expectation (Fig. 4D). However, RNAse treatment of samples prior to EV purification to get rid of extralumenal miRNA was dismissed as a previous report highlighted that Ago2 protects miRNAs against RNAses^67^. This additional step would also complicate clinical application of EVs as medical product.

The miRNA let-7a was used as internal reference to compare Cq values across samples in this study. Although let-7a was identified most suitable for normalisation via NormFinder in our samples, others report that this miRNA performs poorly for other EVs like adipose-derived MSC EVs^68^. In contrast, let-7a was found to be highly abundant in milk across species as well as in plasma or serum of healthy individuals and traveled with EVs rather than soluble factors like Ago2^69–71^, which supports the idea that the optimal reference miRNA might be sample type specific. Nevertheless, we identified characteristic sets of miRNAs enriched or depleted in EVs purified from the blood products CPRP and hypACT (Fig. 4D,E). Interestingly, miRNAs were differentially enriched or depleted in blood product EVs. miR-101 was significantly depleted in both CPRP and hypACT EVs, whereas miR-27a was depleted in CPRP EVs, but strongly enriched in hypACT EVs (Fig. 4D). This indicates that clotting might promote changes in the EV-associated miRNA repertoire, for example via EVs supporting coagulation^72^ or release during or after clotting. Lower levels of particular miRNAs might in fact be favourable, as miR-101 targets Sox9, therefore miR-101 might decrease the regenerative potential of blood products compared to isolated EVs^73^. Nevertheless, the identified miRNA profiles might be influenced by the EV purification approach, therefore applying different procedures to obtain pure EVs for miRNA profiling will substantiate our findings.

Potential miRNA-mRNA target interactions of miRNAs found in CPRP and hypACT EVs were analysed via STRING including functional enrichment analysis and physical relations between protein products of target mRNAs. While these targets included shared clusters, some clusters were specific for either CPRP EVs or hypACT EVs (Fig. 5). A prominent target of miRNAs CPRP EVs is NFκB signaling, whereas IL6 signaling is targeted by hypACT EV miRNAs. Indeed, treating patient-derived chondrocytes with EVs enriched via UC produced a decrease of NFκB levels in response to CPRP EVs and IL6 release was decreased from cells exposed to hypACT EVs enriched via UC only instead UC + SEC + UC as shown in a previous work from our group^30^. The latter might be a concerted result from inhibitory effects on IL6 mRNA mediated by miR-223, miR-142-3p, let-7a and miR-26a which are all found in hypACT EVs and target IL6 with strong experimental evidence according to miRTarBase. In addition, Notch/Delta signaling, which is specifically targeted by CPRP EVs (Fig. 5), was identified as inhibitor of chondrogenic differentiation^74,75^.

Focusing on miRNAs that are enriched in EVs compared to blood products (Fig. 4D,E) revealed two clusters of genes strongly targeted by hypACT EVs (Fig. 6). Among those is SMAD3, which has been identified as risk factor for OA^76^. The stringent selection criteria that were used in this analysis might leave less strongly enriched terms unnoticed, though being potentially relevant.

Recently, a study determined how many miRNA molecules are found per EV^77^. It turned out that only 1 miRNA copy was present in 300 to 1.6 × 10^4^ EVs for an individual viral miRNA, which resulted in the statement that miRNAs are minor or almost irrelevant constituents of EVs. Similarly, we found that an individual miRNA species of our screening panel was present in around 2 × 10^4^ EVs on average. However, blood EVs bear a plethora of different miRNA species. Any EV which does not carry a given miRNA species might carry one or more different ones, totaling up to 1 in 24 or 1 in 379 EVs on average which carries any miRNA in CPRP or hypACT, respectively (Table 1). This stands against the claim that miRNAs would be negligible components of EV cargo. In addition, one might argue that not the absolute amount of a given miRNA is important. Although, the copy number might not be sufficient to substantially modulate a given mRNA with relevant stoichometry, a group of miRNAs targeting the same mRNA or mRNAs of related factors in a given pathway like TGFβ/SMAD signaling could still result in relevant, even disease modifying, effects. Therefore, as miRNAs can target a pathway on multiple levels, effects mediated by miRNAs might be more than the sum of their parts.

Intra-articular injection of isolated (autologous) EVs for cartilage repair might provide more stable outcomes in cartilage repair than blood products. EVs as fraction of blood products are probably less prone to donor variability, which integrates differences in all other blood product constituents as well. Therefore, focusing on EVs and EV-asociated miRNAs might allow a more detailed prediction of therapeutic outcomes.

Although the identified functions encoded in the miRNA repertoire of blood product EVs suggest that they might facilitate cartilage repair, basic questions remain that need to be addressed in animal studies before clinical translation. First of all, the bioavailability and biodistribution within the joint after injection has to be evaluated as EVs need to come in contact with chondrocytes to exert their predicted effects. In the following, therapeutic efficacy and outcomes such as cartilage tissue regeneration and homeostasis, alongside with effects on inflammation in the joint need to be investigated to estimate the clinical potential of EVs in cartilage repair.

In conclusion, EVs from CPRP and hypACT carry diverse miRNA cargos with distinct functional repertoires, which might be sufficient to induce regenerative processes in cartilage and might supersede application of full blood products in OA therapy.

## Supplementary Information

Supplementary Information 1.

Supplementary Table 1.

Supplementary Table 2.

Supplementary Table 3.
