# Supplementary material for: Functional repertoire of EV-associated miRNA profiles after lipoprotein depletion via ultracentrifugation and size exclusion chromatography from autologous blood products
Source: Sci Rep. 2021 Mar 12;11:5823. doi: 10.1038/s41598-021-84234-5 (PMC7955123; doi:10.1038/s41598-021-84234-5)

Functional repertoire of EV-associated miRNA profiles after lipoprotein depletion via ultracentrifugation and size exclusion chromatography from autologous blood products

Alexander Otahal[1], Olga Kuten-Pella[2], Karina Kramer[1], Markus Neubauer[1], Zsombor Lacza[3], Stefan Nehrer[1], Andrea de Luna[1*]

[1] Center for Regenerative Medicine, Department for Health Sciences, Medicine and Research, Danube University Krems, Krems an der Donau, Austria

[2] OrthoSera GmbH, Krems an der Donau, Austria

[3] Department of Sports Physiology, University of Physical Education, Budapest, Hungary

* Corresponding author

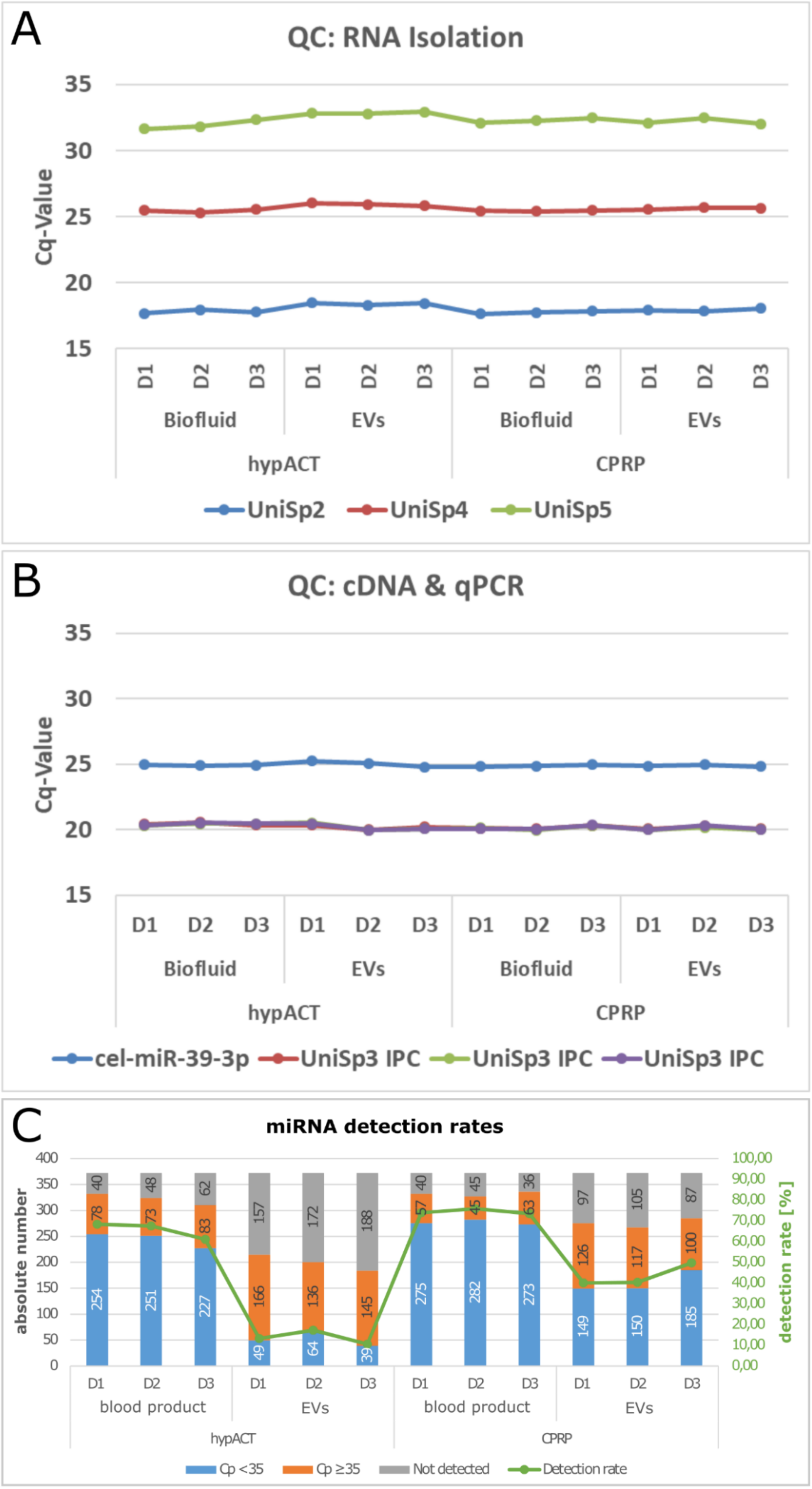

Fig S1. Quality control of RT-qPCR-based miRNA quantification and miRNA detection rates (A) RNA extraction controls, (B) reverse transcription controls. (C) Number of miRNA species identified per sample.

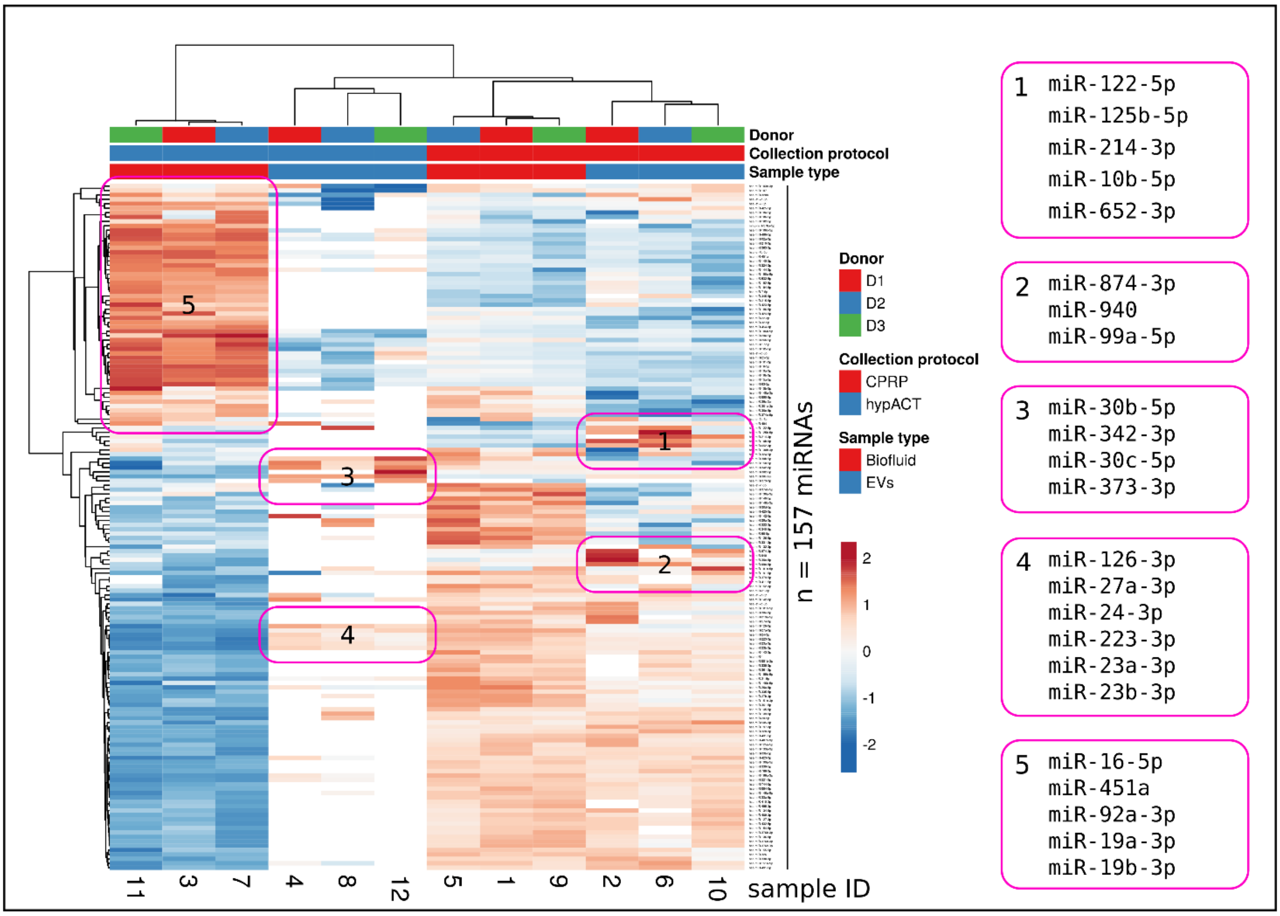

Fig S2. Heat map of ΔCp values, normalised to let-7a. Hierarchical clustering analysis was performed via ClustVis [75] using the default settings, i.e. singular value decomposition with imputation, pearson correlation as the distance metric, and average distance for clustering.

Supplementary data 1 – PHP implementation of Savitzky-Golay algorithm

```
/*
 *
 * Perform size distribution smooting using the Savitzky-Golay polynomial approach
 *
 * @ref statistics4u.info/fundstat_eng/cc_filter_savgolay.html
 * @ref statistics4u.info/fundstat_germ/cc_savgol_coeff.html
 *
 * @param array $a - size distribution as list of concentrations
 * @$neighbours int $neighbours - chosen number of neighbours of target value
 *
 * @return array $smoothed_a - smoothed size distribution
 *
 */
function savitzky_golay_smooth ($a,$neighbours) {

		$neighbours = intval($neighbours);

		if ( is_numeric($neighbours) && $neighbours > 1 && $neighbours < 13 ) {

		//backup
		$smoothed_a = $a;
		//list of coefficients indexed by neighbour parameter
		//np is the number of values used for smoothing
		$coefficients = array(
		2 => array(-3,12,17,12,-3), //np=5
		3 => array(-2,3,6,7,6,3,-2), //np=7
		4 => array(-21,14,39,54,59,54,39,14,21), //np=9
		5 => array(-36,9,44,69,84,89,84,69,44,9,-36), //np=11
		6 => array(-11,0,9,16,21,24,25,24,21,16,9,0,-11), //np=13
		7 => array(-78,-13,42,87,122,147,162,167,162,147,122,87,42,-13,-78), //np=15
		8 => array(-21,-6,7,18,27,34,39,42,43,42,39,34,27,18,7,-6,-21), //np=17
		9 => array(-136,-51,24,89,144,189,224,249,264,269,264,249,224,189,144,89,24,-51,-136), //np=19
		10 => array(-171,-76,9,84,149,204,249,284,309,324,329,324,309,284,249,204,149,84,9,-76,-171), //np=21
		11 => array(-42,-21,-2,15,30,43,54,63,70,75,78,79,78,75,70,63,54,43,30,15,-2,-21,-42), //np=23
		12 => array(-253,-138,-33,62,147,222,287,343,387,422,447,462,467,462,447,422,387,343,287,222,147,62,-33,-138,-253) //np=25
		);
		//list of normalisation factors indexed by neighbour parameter
		$h = array(
		2 => 35,
		3 => 21,
		4 => 231,
		5 => 429,
		6 => 143,
		7 => 1105,
		8 => 323,
		9 => 2261,
		10 => 3059,
		11 => 805,
		12 => 5175
		);

		//define position range
		$positions = range(-$neighbours,$neighbours);

		//perform smoothing
		for ($i=$neighbours;$i<count($a)-$neighbours;$i++) {
			//calculate
			for ($y=0,$j=0;$j<count($coefficients[$neighbours]);$j++) {
				$y += $coefficients[$neighbours][$j]*$a[$i+$positions[$j]];
			}
			//normalize
			$y = $y/$h[$neighbours];
			//set negative value to 0, as there can't be negative particle concentration
			if ( $y < 0 ) {
				$smoothed_a[$i] = 0;
			} else {
				$smoothed_a[$i] = $y;
			}
		}
		} else {
		die("Wrong neighbours parameter for Savitzky-Golay smoothing");
		}
		return $smoothed_a;
}
```

Supplementary data 2 – Full Western Blot scans

Blots in Fig 3A

ApoA1, 10 µg per lane, except PL (20 µg)
10% Bis/Tris SDS-PAGE, non-reducing

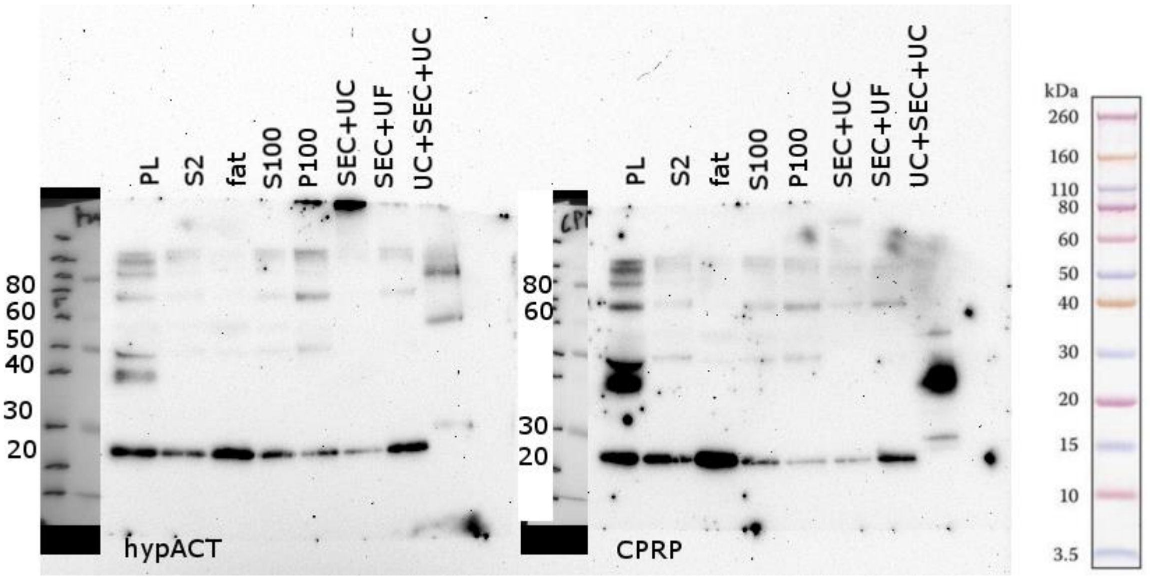

CD9, 10 µg per lane, except PL (20 µg)
10% Bis/Tris SDS-PAGE, non-reducing

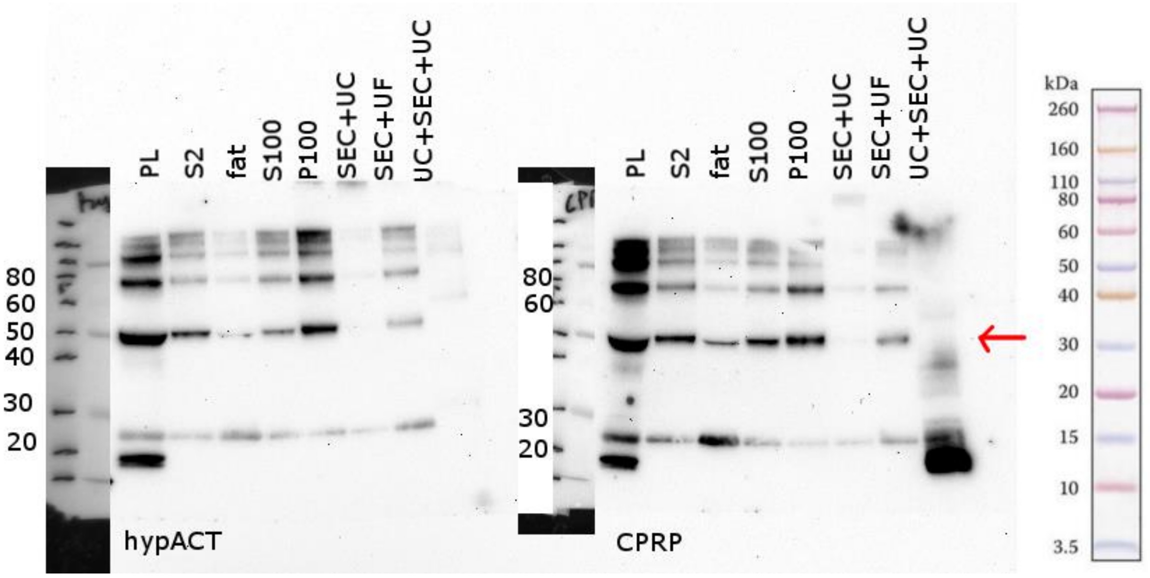

Alix, 10 µg per lane, except PL (20 µg)
4-12% Bis/Tris SDS-PAGE, reducing

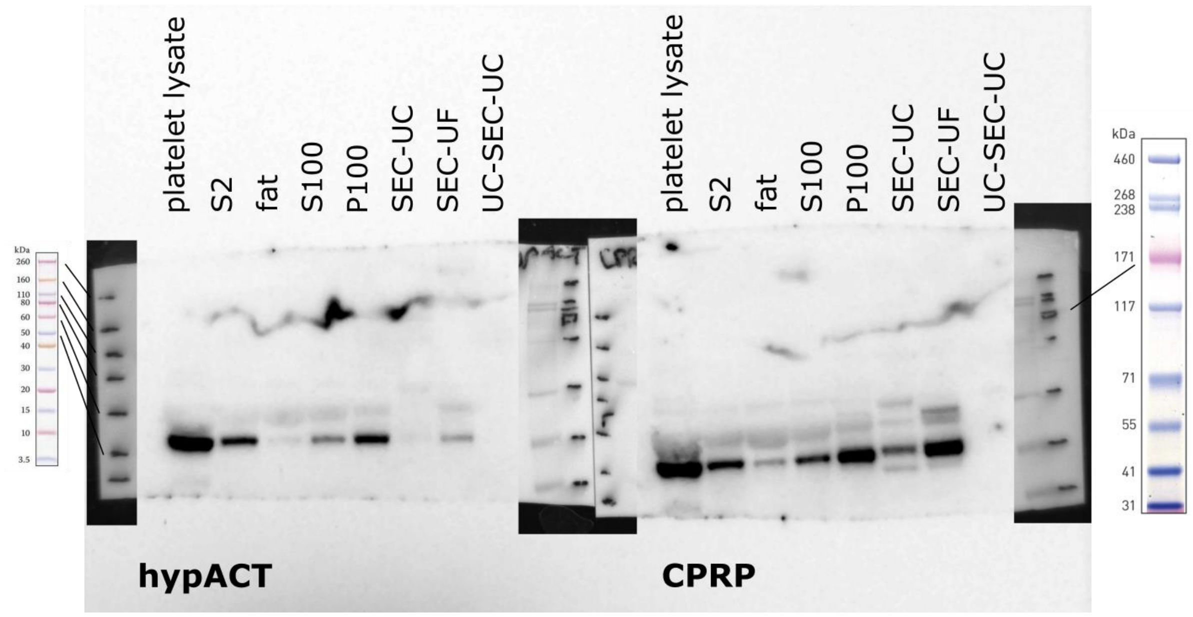

ApoB100, 10 µg per lane, except PL (20 µg)
4-12% Bis/Tris SDS-PAGE, reducing

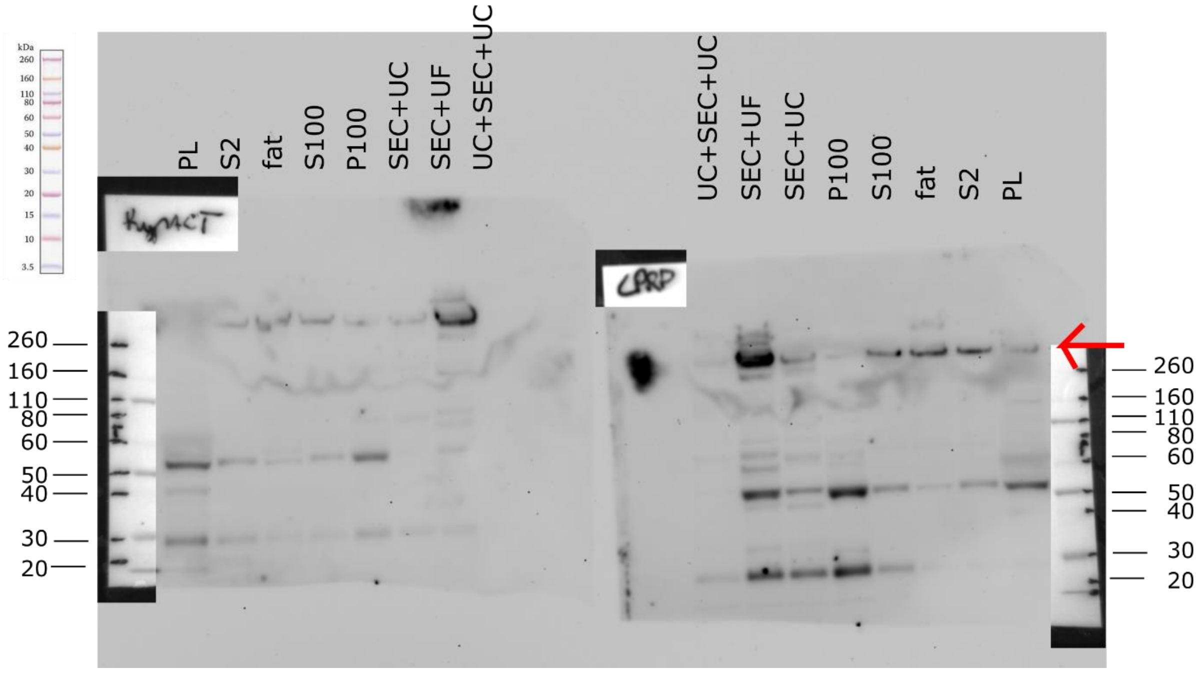

Blots in Fig 3B

CD63, 80 µg per lane, except PL (20 µg)
10% Bis/Tris SDS-PAGE, non-reducing

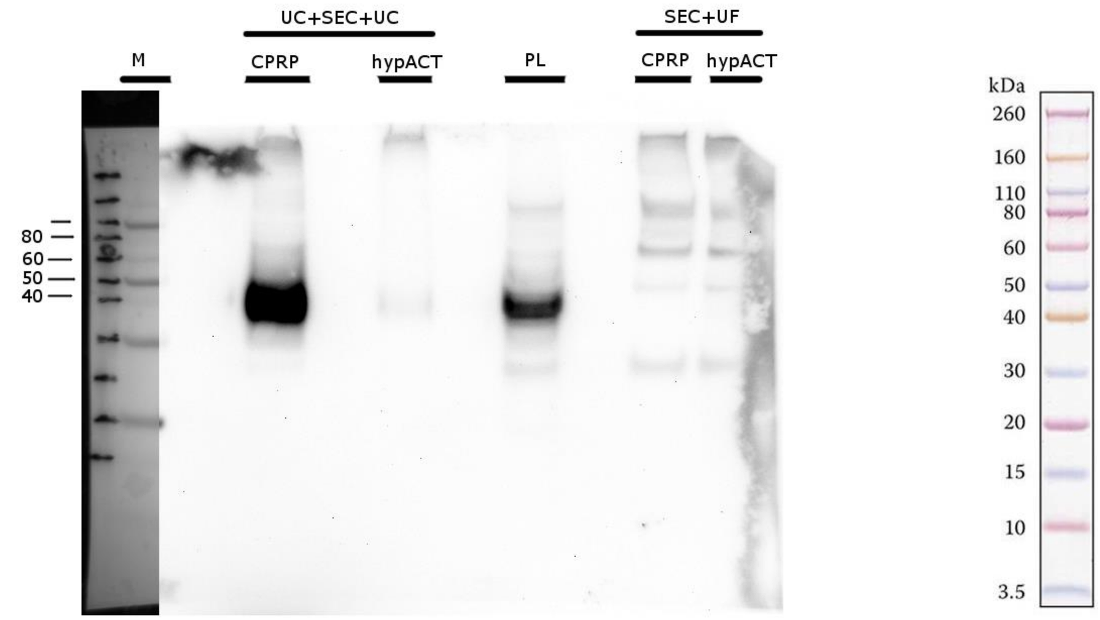

CD9, 80 µg per lane, except PL (20 µg); reprobed after detecting CD63 and washing
10% Bis/Tris SDS-PAGE, non-reducing

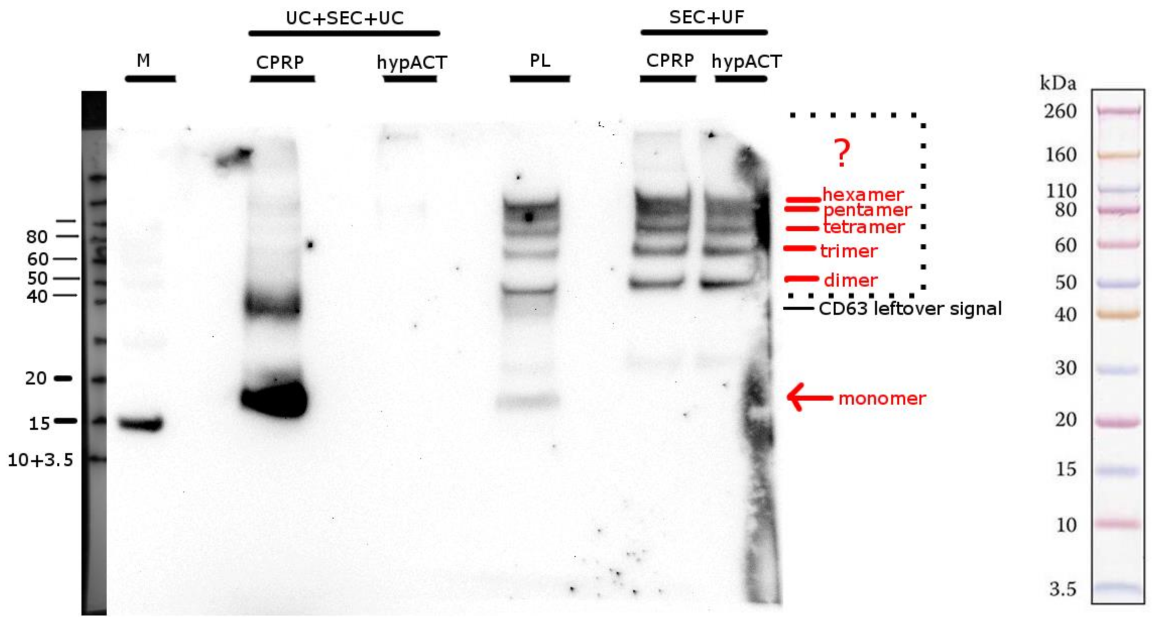

Supplement: Supplementary file 1 — Supplementary Information 1. [file 41598_2021_84234_MOESM1_ESM.pdf]
